# High Platelet-to-Lymphocyte Ratio Predicts Poor Prognosis and Clinicopathological Characteristics in Patients with Breast Cancer: A Meta-Analysis

**DOI:** 10.1155/2017/9503025

**Published:** 2017-08-31

**Authors:** Miao Zhang, Xuan-zhang Huang, Yong-xi Song, Peng Gao, Jing-xu Sun, Zhen-ning Wang

**Affiliations:** ^1^Department of Oncology, The Air Force General Hospital of Chinese PLA, No. 30 Fucheng Road, Haidian District, Beijing City 100142, China; ^2^Department of Chemotherapy and Radiotherapy, The Second Affiliated Hospital and Yuying Children's Hospital of Wenzhou Medical University, 109 Xueyuan West Road, Lucheng District, Wenzhou City 325027, China; ^3^Department of Surgical Oncology and General Surgery, The First Hospital of China Medical University, 155 North Nanjing Street, Heping District, Shenyang City 110001, China

## Abstract

**Background:**

We aimed to evaluate the correlation of platelet-to-lymphocyte ratio (PLR) with prognosis and clinicopathological characteristics of breast cancer.

**Methods:**

The PubMed and Embase databases were searched. Hazard ratio (HR) with 95% confidence interval (CI) was used to summarize disease-free survival (DFS) and overall survival (OS). Odds ratio (OR) was used to summarize tumor clinicopathological characteristics.

**Results:**

High PLR was associated with poor DFS and OS (DFS: HR = 1.47, 95% CI = 1.16–1.85, and Tau^2^ = 0.070; OS: HR = 1.88, 95% CI = 1.27–2.80, and Tau^2^ = 0.192). A Galbraith plot indicated that the studies by Allan et al. and Cihan et al. contributed the heterogeneity of DFS and OS, respectively. There were significant differences in the incidence of high PLR between stage II–IV and stage I groups (OR = 1.86, 95% CI = 1.20–2.90, and Tau^2^ < 0.001), between lymph node-positive and lymph node-negative groups (OR = 1.52, 95% CI = 1.22–1.91, and Tau^2^ =0.014), and between metastasis-positive and metastasis-negative groups (OR = 4.24, 95% CI = 2.73–6.59, and Tau^2^ < 0.001).

**Conclusions:**

Our results indicated that PLR was associated with poor prognosis of breast cancer and adequately predicted clinicopathological characteristics.

## 1. Introduction

Breast cancer is the most frequently diagnosed cancer in women worldwide, accounting for approximately 25% (1.68 million) of all cancer cases [1]. Clinical treatment decisions based on tumor characteristics, patient characteristics, and response to treatment have been widely used for breast cancer and contribute to the decreased mortality [2–4]. Nevertheless, the heterogeneity of breast cancer is characterized by its variable natural course and patients' response to treatment and, for this reason, breast cancer is still the leading cause of cancer death in women, accounting for approximately 15% (521,900) of all cancer deaths worldwide [1].

Several translational medicine studies have reported that the interaction between tumor and host microenvironments, including inflammation and immune response, plays an important role in tumor progression and prognosis [5–9]. Recently, platelet-to-lymphocyte ratio (PLR) has become an attractive, convenient, and cost-effective blood-derived prognostic marker as well as an inflammation-related and immune-related prognostic score to evaluate the prognosis of several solid tumors. Indeed, studies showed that elevated PLR was associated with poor prognosis for colorectal cancer [10, 11]. Moreover, several studies showed that elevated PLR was a good predictor for poor prognosis in gastric cancer and lung cancer [12–14]. However, the relationship between PLR and prognosis of breast cancer is controversial and has not been confirmed [15–22]. In addition, it is not known whether the PLR can predict the clinicopathological characteristics of breast cancer.

To date, no meta-analyses have evaluated the relationship between PLR and clinicopathological characteristics and prognosis in breast cancer. Therefore, the purpose of our study was to evaluate the clinicopathological and prognostic significance of the PLR in breast cancer.

## 2. Materials and Methods

### 2.1. Search Strategy

A systematic literature search of relevant studies was conducted using PubMed and Embase databases, with no restrictions on country of publication or year of publication (up to May 2017). We included English publications because English is the most widely used language worldwide. The main keywords and MeSH terms used were “platelet-to-lymphocyte ratio”, “platelet-lymphocyte ratio”, “platelet to lymphocyte ratio”, “PLR”, “thrombocyte-lymphocyte ratio”, “thrombocyte to lymphocyte ratio”, “breast cancer”, “breast tumor”, “breast neoplasms”, and “breast carcinoma”. We also performed a manual search of the reference lists of the retrieved studies and reviews to identify potential studies.

### 2.2. Eligibility Criteria

The studies that met the following inclusion criteria were (1) those that evaluated patients with breast cancer only; (2) those that measured PLR in the blood of patients with breast cancer; (3) those that evaluated the prognostic values of PLR in breast cancer; and (4) the outcome measures of interest that could be extracted directly or could be calculated from the published data indirectly. And studies were excluded from the meta-analysis if the outcome measures were not reported and could not be calculated from the published data. If several duplicated studies based on the same patient population met the inclusion criteria, only the most informative study was included.

### 2.3. Data Extraction and Quality Assessment

Data extraction and quality assessment of the studies included were performed by two reviewers (Miao Zhang and Xuan-zhang Huang), independently. The following data were extracted: first author, country, year of publication, sample size, patient characteristics, sampling time, cut-off point, follow-up period, clinicopathological characteristics of the tumor, and prognostic value (overall survival [OS] and disease-free survival [DFS]).

We used Newcastle-Ottawa Scale (NOS) criteria to assess the quality of the included studies [23]. Moreover, any disagreements in data extraction and quality assessment were resolved via discussion.

### 2.4. Statistical Analysis

Our meta-analysis was performed according to the Preferred Reporting Items for Systematic Reviews and Meta-analyses (PRISMA) statement (Supplemental File 1: PRISMA Checklist in Supplementary Material available online at https://doi.org/10.1155/2017/9503025) [24]. We used hazard ratios (HR) with corresponding 95% confidence intervals (CI) as effect measures to evaluate the relationship between PLR and breast cancer prognosis. The included studies were retrospective studies, and the PLR level was measured at the time of diagnosis without a follow-up period; thus PLR was used as a variable but not a predictor, and odds ratio (OR) was used as an effect measure when assessing the association between PLR and clinicopathological characteristics. The OR represented the odds of occurrence of an unfavorable event in a high PLR level compared with a low level. In cases in which the HR, OR, and 95% CI could not be extracted directly, they were manually calculated from published data [25, 26]. Then, the log HR, log OR, and corresponding standard error (SE) were used to compute a pooled effect measures. Moreover, subgroup analyses were performed on the basis of the sampling time, sample size, cut-off point, and the status of receptors (estrogen receptor (ER), progesterone receptor (PR), and human epidermal growth factor receptor 2 [HER2]).

The heterogeneity between the studies was calculated using tau-squared (Tau^2^) statistics, *I*^2^ statistics, and Cochran *Q* test [27]. The Tau^2^ statistics represented the real measure of between-study heterogeneity, and the *I*^2^ statistics represented the approximate proportion of total variability that could be attributed to heterogeneity [28]. The heterogeneity was considered statistically significant when the *P* value was <0.05 in the *Q* test and/or *I*^2^ was >50%, and then the random-effect model was used to pool the effect measures; otherwise, a fixed effects model was used. The potential sources of heterogeneity were explored by subgroup analyses and Galbraith plot. Publication bias was evaluated using Begg's and Egger's tests and funnel plot. In addition, in cases of significant publication bias, we performed trim-and-fill analysis to assess the effect of this bias [29–31].

All statistical analyses were performed using Stata software version 12.0 (Stata Corporation, College Station, TX, USA). Two-sided *P* values < 0.05 were considered statistically significant.

## 3. Results

### 3.1. Study Selection and Study Characteristics

**Figure 1:**
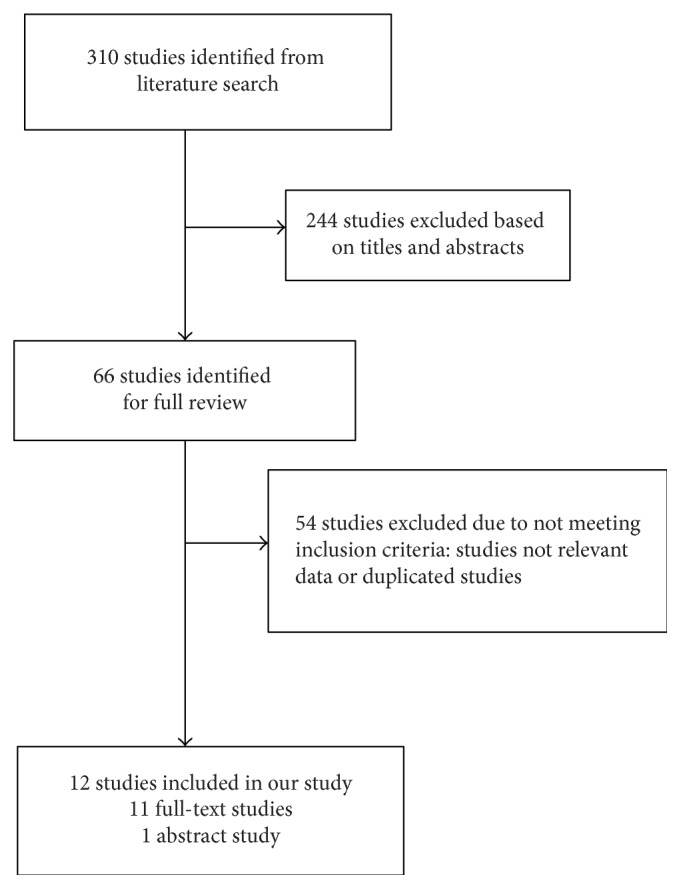
Literature search and study selection.

Three hundred and ten relevant studies were initially identified; of these, 244 studies were excluded after reviewing the titles and abstracts. The full texts of the remaining 66 studies were evaluated. An additional 54 studies were excluded and twelve studies were identified as eligible in the meta-analysis (Figure 1) [15–22, 32–35].

The twelve eligible studies evaluated 5542 patients with breast cancer (median and range of sample size: 668 and 62–1435; average sample size: 462). The studies were performed in Turkey, Malaysia, China, Austria, USA, Japan, Costa Rica, and the United Kingdom. The analysis of the sampling time indicated that nine studies included patients with preoperative PLR [16–22, 32, 33], two studies included patients with preoperative or prechemoradiotherapy PLR [15, 34], and one study did not report the sampling time [35]. Of the eligible studies, eleven studies assessed the relationship between DFS and PLR [15–22, 32, 33, 35], and eight studies assessed the relationship between OS and PLR [16–21, 33, 35]. The detailed study characteristics and study quality are summarized in Table 1.

## 4. PLR and Survival

### 4.1. PLR and DFS

**Figure 2:**
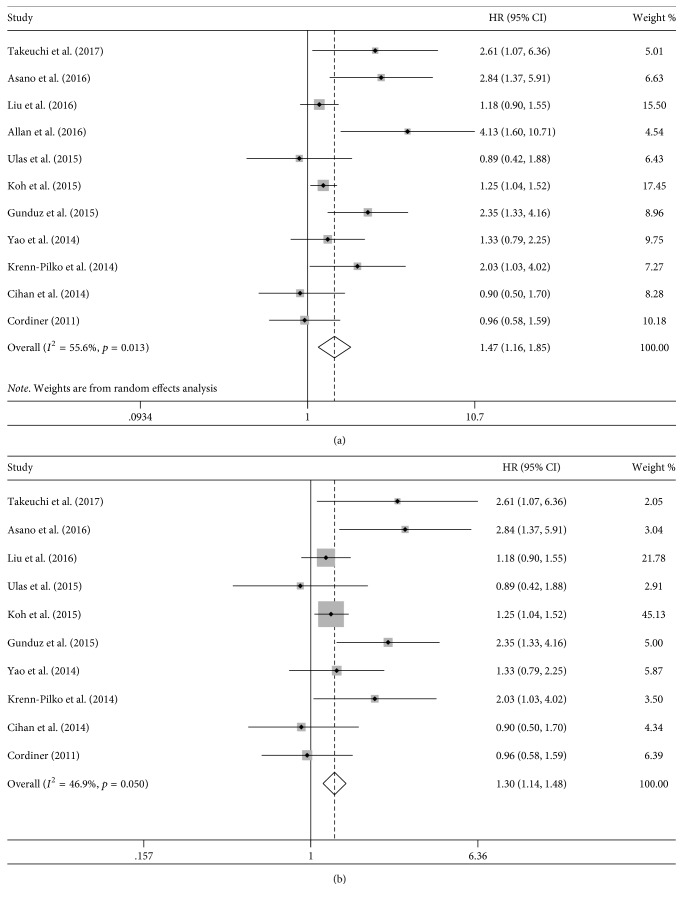
Hazard ratio (HR) with 95% confidence interval (CI) summary for the association between platelet-to-lymphocyte ratio and disease-free survival: (a) including the study by Allan et al.; (b) excluding the study by Allan et al. HR > 1 represents an unfavorable prognosis in the high PLR level compared with the low PLR level.

**Figure 3:**
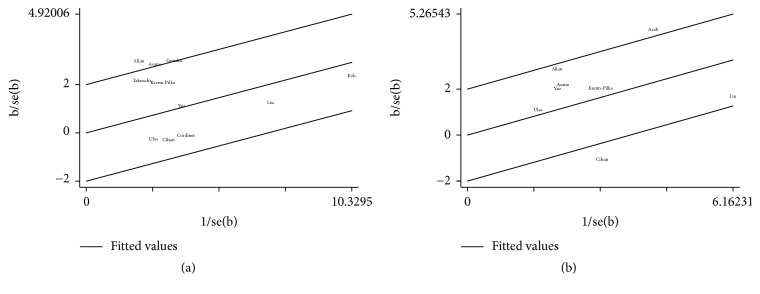
Galbraith plot for exploring the sources of heterogeneity on disease-free survival (a) and overall survival (b).

Eleven studies evaluated the relationship between DFS and PLR [15–22, 32, 33, 35]. Our results indicate that DFS was significantly shorter in breast cancer patients with high PLR level compared with those with low PLR level (HR = 1.47 and 95% CI = 1.16–1.85; Figure 2), with significant heterogeneity (Tau^2^ = 0.070 and *I*^2^ = 55.6%). Galbraith plot showed that the study by Allan et al. [19] might contribute to the heterogeneity (Figure 3); as expected, the heterogeneity decreased (Tau^2^ = 0.046) after this study was excluded and the prognostic value of PLR was confirmed (HR = 1.30 and 95% CI = 1.14–1.48; Figure 2).

The subgroup analysis based on preoperative PLR (HR = 1.66, 95% CI = 1.21–2.27, Tau^2^ = 0.126, and *I*^2^ = 59.0%) and sample size (sample ≥ 400: HR = 1.26, 95% CI = 1.07–1.48, Tau^2^ = 0.001, and *I*^2^ = 1.7%; sample < 400: HR = 1.61, 95% CI = 1.14–2.28, Tau^2^ = 0.142, and *I*^2^ = 62.5%) provided a similar result (Table 2). The subgroup analysis stratified by cut-off point indicated that the prognostic value was significant when the cut-off point was set at ≥180 (HR = 1.99, 95% CI = 1.20–3.31, Tau^2^ = 0.180, and *I*^2^ = 71.8%) and at <180 (HR = 1.27, 95% CI = 1.04–1.56, Tau^2^ = 0.073, and *I*^2^ = 47.6%). Moreover, the subgroup analysis based on the status of the receptor showed that the prognostic effect of PLR on DFS was similar among the patients with positive hormone receptors (ER^+^ or PR^+^) (HR = 1.59, 95% CI = 1.18–2.13, Tau^2^ = 0.021, and *I*^2^ = 16.2%), HER2^−^ (HR = 1.30, 95% CI = 1.07–1.57, Tau^2^ < 0.001, and *I*^2^ = 0.0%), or triple-negative status (HR = 1.39, 95% CI = 1.04–1.86, Tau^2^ < 0.001, and *I*^2^ = 0.0%). And high PLR tended toward a lower effect of DFS on HER2^+^ (HR = 1.29, 95% CI = 0.85–1.96, Tau^2^ = 0.155, and *I*^2^ = 69.2%) and negative hormone receptors (ER^−^ and PR^−^) (HR = 1.14, 95% CI = 0.93–1.41, Tau^2^ < 0.001, and *I*^2^ = 0.0%) breast cancer, although statistical significance was not reached. The detailed results of subgroup analyses are summarized in Table 2.

### 4.2. PLR and OS

**Figure 4:**
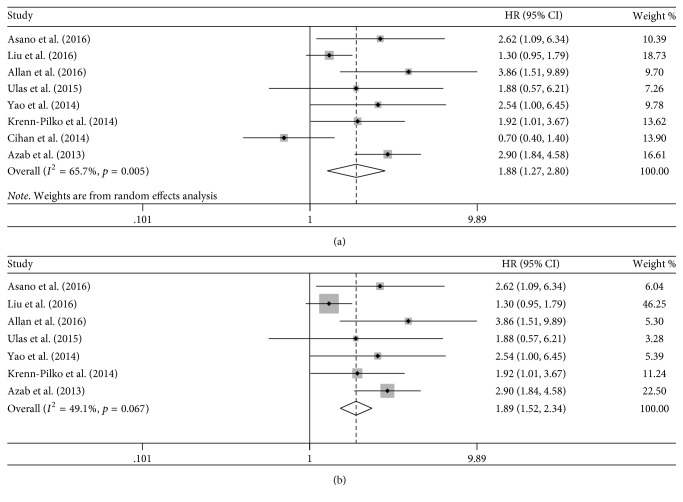
Hazard ratio (HR) with 95% confidence interval (CI) summary for the association between platelet-to-lymphocyte ratio and overall survival: (a) including the study by Cihan et al.; (b) excluding the study by Cihan et al. HR > 1 represents an unfavorable prognosis in the high PLR level compared with the low PLR level.

Eight studies evaluated the relationship between OS and PLR [16–21, 33, 35]. The poor prognosis for OS in patients with breast cancer was indicated by the high level of PLR (HR = 1.88 and 95% CI = 1.27–2.80; Figure 4), with significant heterogeneity (Tau^2^ = 0.192 and *I*^2^ = 65.7%). A Galbraith plot showed that the study by Cihan et al. [17] contributed to the high heterogeneity (Figure 3), and the heterogeneity decreased (Tau^2^ = 0.099) after excluding this study and the prognostic value of PLR was confirmed (HR = 1.89; 95% CI = 1.52–2.34; Figure 4).

Similarly, the study by Cihan et al. [17] contributed to the high heterogeneity in the subgroup analysis of preoperative PLR (HR = 1.71, 95% CI = 1.14–2.57, Tau^2^ = 0.155, and *I*^2^ = 56.5%). After excluding this study, our results also indicated that preoperative high PLR was associated with worse OS without reduced heterogeneity (HR = 1.67, 95% CI = 1.30–2.13, Tau^2^ = 0.062, and *I*^2^ = 32.5%). The subgroup analysis stratified by sample size and cut-off points indicated that the prognostic value of PLR was significant when the sample size was ≥400 (HR = 2.53, 95% CI = 1.79–3.57, Tau^2^ < 0.001, and *I*^2^ = 0.0%) and the cut-off point was set at ≥180 (HR = 2.68, 95% CI = 1.89–3.78, Tau^2^ < 0.001, and *I*^2^ = 0.0%), and high PLR tended toward a worse OS when the sample size was <400 (HR = 1.60, 95% CI = 0.93–2.75, Tau^2^ = 0.223, and *I*^2^ = 65.3%) and the cut-off point was <180 (HR = 1.46, 95% CI = 0.92–2.31, Tau^2^ = 0.135, and *I*^2^ = 53.0%). We could not perform a subgroup analysis on the basis of the receptor status owing to the limited number of studies.

## 5. PLR and Clinicopathologic Characteristics

Our results indicated a significantly higher incidence of high levels of PLR in the stage II–IV group relative to the stage I group (OR = 1.86, 95% CI = 1.20–2.90, Tau^2^ < 0.001, and *I*^2^ = 0.0%). There were significant differences in the incidence of high levels of PLR between the lymph node-positive and lymph node-negative groups (OR = 1.52, 95% CI = 1.22–1.91, Tau^2^ = 0.014, and *I*^2^ = 13.9%) and between the metastasis-positive and metastasis-negative groups (OR = 4.24, 95% CI = 2.73–6.59, Tau^2^ < 0.001, and *I*^2^ = 0.0%). We could not assess the association between tumor pT category and PLR level owing to the limited number of studies.

On the basis of the degree of tumor differentiation, our results indicated a nonsignificant association between the degree of differentiation and the PLR level (poor differentiation versus moderate/high differentiation, OR = 1.15, 95% CI = 0.86–1.54, and *I*^2^ = 0.0%; poor/moderate differentiation versus high differentiation, OR = 1.56, 95% CI = 0.78–3.14, and *I*^2^ = 58.2%). Moreover, there was a significant difference in the incidence of high levels of PLR between HER2^+^ and HER2^−^ status (OR = 1.54, 95% CI = 1.11–2.14, Tau^2^ < 0.001, and *I*^2^ = 0.0%), but not between other receptor statuses (ER^+^ versus ER^−^: OR = 1.08, 95% CI = 0.69–1.72, Tau^2^ = 0.128, and *I*^2^ = 60.7%; PR^+^ versus PR^−^: OR = 0.88, 95% CI = 0.69–1.13, Tau^2^ < 0.001, and *I*^2^ = 0.0%).

## 6. Assessment of Publication Bias

**Figure 5:**
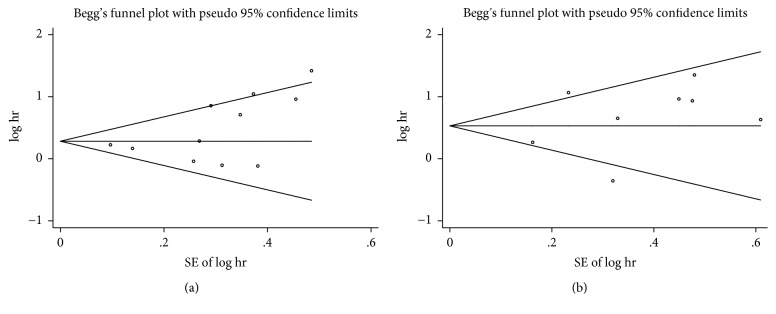
Funnel plots assessing publication bias for disease-free survival (a) and overall survival (b).

Begg's and Egger's tests showed no evidence of publication bias in our study (DFS: Begg's test = 0.09 and Egger's test = 0.11; OS: Begg's test = 0.71 and Egger's test = 0.37). Furthermore, the absence of publication bias was confirmed with a funnel plot (Figure 5). A Galbraith plot showed that the study by Cihan et al. [17] and Allan et al. [19] generated high heterogeneity to the pooled analysis of OS and DFS, respectively (Figure 3). The exclusion of the study could increase the statistical power and reduced heterogeneity.

## 7. Discussion

The present study assessed the association between the prognostic and clinicopathological values and PLR levels by analyzing 5542 cases of patients with breast cancer. Our results indicated that high PLR level was associated with poor prognosis, including OS and DFS. Moreover, we found an association between PLR and clinicopathological characteristics, including tumor stage, lymph node metastasis, and distant metastasis, suggesting a solid basis for breast tumor staging.

Tumor proliferation, invasion, angiogenesis, and metastasis are affected by the host inflammation and immune response in the tumor microenvironment [36–39]. Moreover, our results indicate that PLR can be used as a prognostic factor for breast cancer. Although the underlying causes and mechanisms have not been fully elucidated, there are several possible explanations. A high PLR reflects a decreased number of platelets and/or an increased number of lymphocytes. The platelet may reflect an inflammatory process and play an important role in tumor progression. Bodies of evidence have shown that platelets could influence the metastatic potential of tumor cells via several biological pathways. First, platelets could secrete cellular growth factors including platelet-derived growth factor, vascular endothelial growth factor, transforming growth factor beta, and platelet factor 4, which could stimulate tumor angiogenesis and growth [40–42]. Second, platelets could contribute to the stable adhesion of tumor cells to endothelium and transmigration of tumor cells out of the vasculature. Third, platelets could also enhance tumor stroma formation by promoting the migration of inflammatory cells. Furthermore, platelets could facilitate tumor cell metastasis by impeding the clearance of tumor cells by cell-mediated immunity [43–45]. Thus, a high platelet count was correlated with a poor prognosis.

Numerous studies have demonstrated that lymphocytes play a crucial role in tumor immune surveillance, which suppresses tumor progression and metastasis [39, 46]. Their cytotoxic activity and induction of apoptosis in tumor cells by lymphocytes could control tumor growth [47]. Clinical data have shown that high tumor-infiltrating lymphocytes are associated with favorable prognosis in breast cancer [48, 49]. Accordingly, Mao et al. and Seo et al. reported that tumor-infiltrating lymphocytes could predict the response to neoadjuvant and adjuvant therapy [50, 51]. In addition, lymphopenia may present the status of deficiency of the immune system caused by tumor cells. Therefore, high PLR, with a high platelet count and/or low lymphocyte count, resulted in a low antitumor activity and poor prognosis.

In our study, the prognostic effect of PLR on DFS was significant in patients with ER^+^ or PR^+^ breast cancer. However, PLR could not significantly predict prognosis in patients with ER^−^ and PR^−^ breast cancer. To date, it is not clear whether the prognostic value of the PLR varies among the subpopulations stratified by receptor status. Ulas et al. reported that the PLR has no effect on DFS or OS in HER2^+^ breast cancer [18]. However, Gunduz et al. showed that PLR was associated with DFS in HER2^+^ breast cancer [32]. These contradictory results may be attributed to the ER/PR status. Indeed, Koh et al. thoroughly evaluated the association between PLR and mortality by breast cancer subtype and demonstrated that elevated PLR was associated with an increased risk of mortality in patients with ER^+^ or PR^+^ and HER2^+^ breast cancer but not in patients with ER^−^ or PR^−^ and HER2^+^ breast cancer [15]. Therefore, further studies are needed to evaluate the prognostic value of PLR in different subpopulations stratified by receptor status.

Most studies primarily focused on the prognostic value of PLR in breast cancer and only a few studies explored its association with clinicopathological characteristics. Therefore, both the prognostic value and the clinicopathological value of PLR were explored in this study. Our results indicated that high levels of PLR could predict clinicopathological characteristics, such as tumor stage, lymph node metastatic status, and distant metastatic status. Recent strategies for the treatment of breast cancer have accounted for the status of receptors, including ER, PR, and HER2. Therefore, we explored whether PLR was associated with the status of receptors. Our results indicated no significant associations between PLR and receptor status. Moreover, we did not assess whether the few number of studies included would impact these results. Therefore, future studies should thoroughly evaluate the association between PLR and receptor status.

For the clinical use of PLR, the optimal cut-off value may be the main concern for physicians. Unfortunately, for predicting prognosis in patients with breast cancer, this value has not been determined. Krenn-Pilko et al. reported that high PLR was associated with poor prognosis using a cut-off value of 292 [16] and Gunduz et al. reported that high PLR was associated with poor prognosis using a cut-off value of 200 [32]. However, Ulas et al., Yao et al., and Cihan et al. reported that PLR could not be used as a prognostic factor for breast cancer using a cut-off value of 161, 107, and 160, respectively [17, 18, 33]. Our subgroup analysis stratified by cut-off point indicated that the HR for OS was significant when the cut-off point was set at ≥180 but not at <180. Moreover, Azab et al. reported that the HR for the 4th quartile of PLR (cut-off point > 185) compared with the 1st quartile of PLR (cut-off point < 109) was 3.68 (95% CI = 1.74–7.77; *P* = 0.001) and the HR was 1.02 (95% CI = 1.015–1.027; *P* < 0.001) for every 10-unit increase in PLR, considering PLR as a continuous variable [34]. Therefore, a high cut-off point for PLR may be more valuable and useful. However, a higher cut-off value may result in the omission of a substantial number of patients in clinical practice. Indeed, Azab et al. reported that <10% of patients were grouped into the subset of PLR > 292 and the prognostic value of PLR might be different between the lymphopenia subset and the normal lymphocyte count subset, despite using the same cut-off value [34]. Therefore, further studies should be conducted to define the optimal cut-off value of PLR for future individual treatments.

Although a previous meta-analysis assessed the prognostic value of PLR for breast cancer, the number of included studies was small and the cut-off value of PLR was not assessed [52]. Our study had several obvious advantages. First, our study included more eligible studies and so our results were more robust and powerful. Second, our studies evaluated the impact of cut-off value of PLR via subgroup analyses based on cut-off value. Moreover, our study assessed the associations between receptor status and PLR, and subgroup analyses were performed by the receptor status.

Several limitations should be addressed. First, this retrospective study is based on published data from the studies included. Therefore, some HR values were calculated from published data in case the studies evaluated did not provide them directly. Second, there was a considerable degree of heterogeneity among the studies included and could not be explained and eliminated completely. Our subgroup analysis indicated that sample size might be a source of heterogeneity. After including sample sizes ≥400, the heterogeneity among the studies decreased for both DFS (overall: Tau^2^ = 0.070 and *I*^2^ = 55.6%; ≥400: Tau^2^ = 0.001 and *I*^2^ = 1.7%) and OS (overall: Tau^2^ = 0.192 and *I*^2^ = 65.7%; ≥400: Tau^2^ < 0.001 and *I*^2^ = 0.0%). A possible reason was that a large sample size could improve the reliability and reduce the variability of the results. For the OS, the different cut-off values of PLR also contributed to substantial heterogeneity (overall: Tau^2^ = 0.192 and *I*^2^ = 65.7%; ≥180: Tau^2^ < 0.001 and *I*^2^ = 0.0%). For individual studies, the Galbraith plot indicated that the studies by Cihan et al. [17] and Allan et al. [19] contributed for the heterogeneity of OS and DFS, respectively. Indeed, the heterogeneity was reduced after the removal of these studies (OS without the data from Cihan et al.: Tau^2^ = 0.099; DFS without the data from Allan et al.: Tau^2^ = 0.046), and the results remained significant. The remaining unexplained heterogeneity may have been caused by differences in the tumor characteristics (i.e., receptor status and TNM stage), population demographics, methodology, and other confounding factors. Heterogeneity did not affect or dominate our results. Third, PLR may be affected by several important factors, including bacterial diseases, viral infections, and oral drugs. These inherently confounding factors may not be controlled completely and therefore may underestimate the prognostic value of PLR in breast cancer.

## 8. Conclusions

Our results indicated that high PLR was significantly associated with poor prognosis of breast cancer and adequately predicted clinicopathological characteristics. Future high-quality and well-designed studies are needed to identify the optimal cut-off value of PLR and improve the clinical utility of PLR.

## Supplementary Material

Supplemental File 1: PRISMA Checklist.

## Figures and Tables

**Table 1:** Baseline characteristics and design variables of eligible studies.

Article	Country	Year	Number of patients	Age: mean ± SD/median (range)	Follow-up: mean ± SD/median (range)	Cut-off point	Outcome	Study quality
Takeuchi et al. [22]	Japan	2017	296	NR	Mean: 41	162.28	DFS: 2.61 (1.07–6.36)	7
Asano et al. [20]	Japan	2016	177	NR	Range: 1.2–72	150	DFS: 2.84 (1.37–5.91) OS: 2.62 (1.09–6.34)	7
Liu et al. [21]	China	2016	318	Median: 45 (19–71)	Median: 58.1 (5.9–136.1)	147	DFS: 1.18 (0.9–1.55) OS: 1.30 (0.95–1.79)	6
Allan et al. [19]	Costa Rica	2016	172	Mean: 54.2 ± 12.7	Median: 79.3 (2–90)	250	DFS: 4.13 (1.60–10.71) OS: 3.86 (1.51–9.89)	7
Ulas et al. [18]	Turkey	2015	187	Mean: 51.4 ± 10.7	Median: 26.0 (6.0–84.0)	161.28	DFS: 0.89 (0.42–1.88) OS: 1.88 (0.57–6.21)	7
Koh et al. [15]	Malaysia	2015	1435	Median: 52	NR	185	DFS: 1.25 (1.04–1.52)	6
Gunduz et al. [32]	Turkey	2015	62	Median: 52 (24–73)	Median: 48.4	200	DFS: 2.35 (1.33–4.16)	6
Yao et al. [33]	China	2014	608	Mean: 52.4 ± 10.8; range: 26–86	Median: 42 (8–62)	107.64	DFS: 1.33 (0.79–2.25) OS: 2.54 (1.00–6.45)	6
Krenn-Pilko et al. [16]	Austria	2014	793	Mean: 57.9 ± 12.2	Median: 98 ± 29.2	292	DFS: 2.03 (1.03–4.02) OS: 1.92 (1.01–3.67)	7
Cihan et al. [17]	Turkey	2014	350	Mean: 55.2 ± 0.3; range: 26–86	Range: 0.3–112	160	DFS: 0.90 (0.50–1.70) OS: 0.70 (0.40–1.40)	6
Azab et al. [34]	USA	2013	437	Mean: 63.6	Mean: 60 ± 0.7	185	OS: 2.90 (1.84–4.58)	7
Cordiner et al. [35]	UK	2011	707	NR	Median: 48.1	NR	DFS: 0.96 (0.58–1.59)	NR

*Note.* DFS: disease-free survival; NR: not reported; OS: overall survival; PLR: platelet to lymphocyte ratio; SD: standard deviation; UK: United Kingdom; USA: United States of America.

**Table 2:** Results of subgroup analyses for prognostic significance of platelet to lymphocyte ratio.

	Disease-free survival	Overall survival
*Overall*	HR = 1.47 [1.16–1.85], Tau^2^ = 0.070, *I*^2^ = 55.6%	HR = 1.88 [1.27–2.80], Tau^2^ = 0.192, *I*^2^ = 65.7%
	Without Allan: HR = 1.30 [1.14–1.48], Tau^2^ = 0.046, *I*^2^ = 46.9%	Without Cihan: HR = 1.89 [1.52–2.34], Tau^2^ = 0.099, *I*^2^ = 49.1%
*Sampling time*		
Preoperative	HR = 1.66 [1.21–2.27], Tau^2^ = 0.126, *I*^2^ = 59.0%	HR = 1.71 [1.14–2.57], Tau^2^ = 0.155, *I*^2^ = 56.5%
	Without Allan: HR = 1.53 [1.13–2.06], Tau^2^ = 0.090, *I*^2^ = 52.5%	Without Cihan: HR = 1.67 [1.30–2.13], Tau^2^ = 0.062, *I*^2^ = 32.5%
*Sample size*		
≥400	HR = 1.26 [1.07, 1.48], Tau^2^ = 0.001, *I*^2^ = 1.7%	HR = 2.53 [1.79, 3.57], Tau^2^ < 0.001, *I*^2^ = 0.0%
<400	HR = 1.61 [1.14–2.28], Tau^2^ = 0.142, *I*^2^ = 62.5%	HR = 1.60 [0.93–2.75], Tau^2^ = 0.227, *I*^2^ = 65.3%
	Without Allan: HR = 1.47 [1.06–2.03], Tau^2^ = 0.099, *I*^2^ = 55.8%	Without Allan: HR = 1.32 [0.80–2.16], Tau^2^ = 0.130, *I*^2^ = 53.9%
*Metastatic status*		
M0	HR = 1.86 [1.18–2.92], Tau^2^ = 0.192, *I*^2^ = 65.0%	HR = 1.62 [1.25–2.08], Tau^2^ = 0.079, *I*^2^ = 39.1%
	Without Allan: HR = 1.63 [1.06–2.50], Tau^2^ = 0.129, *I*^2^ = 57.9%	/
*Cut-off point*		
≥180	HR = 1.99 [1.20–3.31], Tau^2^ = 0.180, *I*^2^ = 71.8%	HR = 2.68 [1.89–3.78], Tau^2^ < 0.001, *I*^2^ = 0.0%
	Without Allan: HR = 1.68 [1.07, 2.64], Tau^2^ = 0.103, *I*^2^ = 64.5%	
<180	HR = 1.27 [1.04–1.56], Tau^2^ = 0.073, *I*^2^ = 47.6%	HR = 1.46 [0.92–2.31], Tau^2^ = 0.135, *I*^2^ = 53.0%
	/	Without Cihan: HR = 1.51 [1.14–1.99], Tau^2^ = 0.033, *I*^2^ = 18.1%
*Receptor status*		
Her2+	HR = 1.29 [0.85–1.96], Tau^2^ = 0.155, *I*^2^ = 69.2%	/
Her2−	HR = 1.30 [1.07–1.57], Tau^2^ < 0.001, *I*^2^ = 0.0%	/
HR+	HR = 1.59 [1.18, 2.13], Tau^2^ = 0.021, *I*^2^ = 16.2%	/
HR−	HR = 1.14 [0.93–1.41], Tau^2^ < 0.001, *I*^2^ = 0.0%	/
TNBC	HR = 1.39 [1.04–1.86], Tau^2^ < 0.001, *I*^2^ = 0.0%	/

*Note.* HER2: human epidermal growth factor receptor; HR: hazard ratio; HR+: positive hormone receptor; HR−: negative hormone receptor; M0: no-metastasis; TNBC: triple-negative breast cancer; “/” symbol: no results due to insufficient studies.
